# Multifaceted academic detailing program to increase pharmacotherapy for alcohol use disorder: interrupted time series evaluation of effectiveness

**DOI:** 10.1186/s13722-016-0063-8

**Published:** 2016-09-15

**Authors:** Alex H. S. Harris, Thomas Bowe, Hildi Hagedorn, Andrea Nevedal, Andrea K. Finlay, Risha Gidwani, Craig Rosen, Chad Kay, Melissa Christopher

**Affiliations:** 1 Center for Innovation to Implementation, Health Services Research and Development, VA Palo Alto Health Care System, Menlo Park, CA USA; 2 Center for Chronic Disease Outcomes Research, Health Services Research and Development, VA Minneapolis Health Care System, Minneapolis, MN USA; 3 Pharmacy Benefits Management, Academic Detailing Program, VA San Diego Health Care System, San Diego, CA USA

**Keywords:** Pharmacotherapy, Medication assisted treatment, Alcohol use disorder, Quality improvement, Implementation, Guideline adherence, Practice guidelines

## Abstract

**Background:**

Active consideration of effective medications to treat alcohol use disorder (AUD) is a consensus standard of care, yet knowledge and use of these medications are very low across diverse settings. This study evaluated the overall effectiveness a multifaceted academic detailing program to address this persistent quality problem in the US Veterans Health Administration (VHA), as well as the context and process factors that explained variation in effectiveness across sites.

**Methods:**

An interrupted time series design, analyzed with mixed-effects segmented logistic regression, was used to evaluate changes in level and rate of change in the monthly percent of patients with a clinically documented AUD who received naltrexone, acamprosate, disulfiram, or topiramate. Using data from a 20 month post-implementation period, intervention sites (n = 37) were compared to their own 16 month pre-implementation performance and separately to the rest of VHA.

**Results:**

From immediately pre-intervention to the end of the observation period, the percent of patients in the intervention sites with AUD who received medication increased over 3.4 % in absolute terms and 68 % in relative terms (i.e., 4.9–8.3 %). This change was significant compared to the pre-implementation period in the intervention sites and secular trends in control sites. Sites with lower pre-implementation adoption, more person hours of detailing, but fewer people detailed, had larger immediate increases in medication receipt after implementation. The average number of detailing encounters per person was associated with steeper increases in slope over time.

**Conclusions:**

This study found empirical support for a multifaceted quality improvement strategy aimed at increasing access to and utilization of pharmacotherapy for AUD. Future studies should focus on determining how to enhance the programs effects, especially in non-responsive locations.

## Background

Both psychosocial and pharmacological treatments for alcohol use disorder (AUD; formerly abuse and dependence) have been found to be effective in reducing symptoms and improving functioning in diverse patient populations [1–5]. In the United States (US), 16.6 million people over 18 years of age in 2013 (7.0 %) met diagnostic criteria for AUD yet only 1.3 million (7.8 %) received any formal treatment [6]. Even within the Veterans Health Administration (VHA), the largest integrated healthcare system in the US with a well-developed system of 220 specialty addiction programs, the treatment rate among the roughly 400,000 patients clinically diagnosed with an AUD in fiscal year 2013 (6.8 % of all VA patients) was only 32 %. Clearly, developing and evaluating strategies to improve access to and engagement in effective treatments for AUD is of utmost importance [7].

Several medications are US Federal Drug Administration (FDA)-approved for the treatment of AUD, and/or have support of effectiveness from high-quality meta-analysis, namely naltrexone, acamprosate, disulfiram, and topiramate. These medications can be prescribed and managed in diverse clinical settings [1–3], allowing patients more options regarding the type and location of their AUD treatment, thus potentially increasing access and treatment engagement. However among both providers and patients, knowledge and use of these medications to treat AUD in the US is very low and variable [8–13]. In VHA, low and variable utilization of these medications for AUD persists even though all of them are on the national formulary and supported by VHA clinical practice guidelines and policies [14]. The VA Uniform Mental Health Services Handbook states that evidence-based pharmacotherapy is to be offered and available to all patients diagnosed with AUD if not medically contraindicated [14]. However, among the VHA patients diagnosed with AUD in FY13, only 5.8 % received evidenced-based pharmacotherapy. Even among Veterans seeking treatment in one of VHA’s specialty addiction treatment programs, only 9.9 % received medication treatment for AUD [9, 15].

Not only is the overall rate of medication receipt low for patients with AUD in VHA, there is substantial facility-level variability [9, 15]. The rate of pharmacotherapy for AUD among Veterans who received SUD specialty care in FY13 ranged from 0 to 21 % across facilities. Low prescribing rates and significant variation across facilities suggests that significant gaps exist in patients’ access to these medications, especially in some locations. The existence of facilitative formulary and clinical policies, as well as near real time performance monitoring [16], may be necessary but has not been sufficient to adequately improve performance on this standard of care.

To address this substantial and persistent quality gap, VHA’s Office of Patient Care Services funded the Academic Detailing for Mental Health Initiatives Program that developed and executed campaigns to improve implementation of pharmacological treatment for several mental health and addictive disorders, including tobacco dependence, schizophrenia, depression, posttraumatic stress disorder, and most recently, and the focus of this study, AUD. In 2013, this quality improvement program sent clinical pharmacy specialists (“academic detailers”) to clinical settings within two western VHA networks. The academic detailers strove to educate, motivate, and enable key health care providers to identify and address the spectrum of hazardous alcohol use, especially to facilitate more active consideration of pharmacological treatment options for AUD. The effectiveness of the AUD campaign of the Academic Detailing Program, and the context and process factors that might be associated with its effectiveness, have not been rigorously evaluated. Thus, the goal of the current study is to evaluate this multifaceted implementation strategy as means to increase utilization of pharmacotherapy for AUD, as well as to provide data that might inform and enhance the ongoing expansion of academic detailing programming across VHA.

## Methods

### The academic detailing program

The primary goal of the Academic Detailing AUD Campaign was to increase the proportion of patients with AUD who were receiving treatment with naltrexone, acamprosate, disulfiram, or topiramate. Simple academic detailing interventions have been shown to have small but significant effects on prescribing practices [17]. However the approach evaluated here was more elaborate and multifaceted than typical academic detailing or educational outreach interventions [17]. Aspects of the program were similar to an external facilitation model where barriers and problems are iteratively identified and addressed in real time [18, 19]. No firm standard existed for the intended number of detailing encounters per site. However given the positive relationship between number of educational outreach visits by pharmaceutical representatives and sales [20], multiple visits were attempted, especially in sites with persistent non-response. As such, this program was akin to a hybrid academic detailing/audit and feedback/external facilitation intervention [19, 21]. Another unique and key feature of the program was the implementation of a real-time informatics dashboard so clinicians and detailers could identify patients who might be candidates for the medications (e.g., recent diagnosis of AUD, not currently receiving medications), as well as to assess site-level performance. Audit and feedback has been shown to be more effective when baseline performance is low, when feedback is given more than once, is given verbally and in writing, and when coupled with support and a clear action plan [17], which were all features of this program.

Table 1 outlines the major activities of the program. After Identifying leadership partners and establishing buy-in (Step 1), detailers identified clinical staff with a high volume of patients with AUD (Step 2), and detailing encounters were requested with these priority clinical staff (Step 3). The goal then was to build rapport and engage staff in discussions about evidence-based medications and other AUD treatment options (Step 4), iteratively identify barriers (Step 5), and provide additional resources including a real-time audit and feedback and case finding tool (Steps 6 and 7). Importantly, the detailers sought a commitment from staff to try to increase use of evidence-based medications for AUD (Step 8), and continued to monitor and problem solve if needed (Step 9).

**Table 1:** Features and methods of academic detailing for mental health initiatives program

Identify leadership partners and establish buy-in: Meeting were requested and conducted with key clinical and pharmacy leadership to explain the goals and methods of the initiative, as well as address concerns and high-level barriers
Identify clinical staff with a high volume of patients with AUD in both primary care and mental health/addiction specialty care: using administrative data, the program identified staff with large numbers of AUD patients
Request detailing encounters with these priority providers, citing the previously established leadership support and enthusiasm
Build relationships with priority providers, engaging them in discussions about evidence-based medications and other practices (e.g., brief intervention for risky drinking): the goal was repeated visits to establish rapport and perceived value by the clinicians who received the resources and services
Explore barriers to prescribing the medications and applying the practices
Examine and address knowledge and beliefs about the supporting evidence
Address knowledge and misunderstandings about policies and scope of competence (e.g., belief that policy prohibits primary care clinicians from prescribing naltrexone or other AUD medications)
Identify and problem-solve structural or logistical barriers (e.g., local policy or practice, staffing medication management visits.)
Introduce the providers to additional resources and tools including patient education tools, and pocket cards with FAQs
Introduce real-time, electronic medical record-integrated audit and feedback tools to identify actionable patients
Seek a commitment from the clinician to try to increase prescribing of the medications
Monitor progress and check back periodically for additional education, barrier identification, problem solving, and feedback. Monitor clinical targets (e.g., the number of patients receiving medications for AUD) and clinician use of the informatics tools, especially the case-finding dashboards. If the targeted prescribing behavior was unaffected, the next detailing session would again explore barriers, problem solve, and motivate clinicians

Academic detailers were recruited and selected with preference for previous training experience in mental health, board certification as pharmacotherapy specialists, post graduate residency training, and strong communication skills assessed during interview process. Due to the clinical service content of the detailing encounters, candidates were sought who had provided direct patient care as part of their previous professional experience with prescribing authority under a scope of practice. The Department of Veteran’s Affairs then contracted Alosa Foundation, a nonprofit organization that develops and implements academic detailing programs to improve prescribing, to provide a basic skills course in academic detailing to the six clinical pharmacists who were selected for VA facilities in California, Nevada and the Pacific Islands. In addition to Academic Detailing Basic Skills workshop, Motivational Interviewing training, adapted for use within the context of a provider outreach visit as opposed to a clinical treatment with a patient population, was another important aspect of detailer training. Campaign specific training on AUD was conducted through virtual seminars and one 3-day face-to-face conference, in which subject matter experts assisted in structuring the AUD campaign timeline and targeted goals.

From April to December 2013, the AUD campaign of the Academic Detailing Program was implemented in two western VA networks, including over 37 medical centers and community-based outpatient clinics. Detailing sessions included predominantly in-person one-on-one sessions, in-person group sessions, and educational in-service sessions, with additional follow-up conducted by email, teleconference, and telephone. A wide variety of staff received detailing encounters including psychologists, psychiatrists, nurses, social workers, clinical pharmacists, primary physicians, clinical managers and other administrators.

### Analysis of overall effectiveness

We evaluated the overall effectiveness of the program using a multi-site, interrupted time series design. The main outcome for the evaluation was the monthly proportion of patients with a clinical encounter in which AUD was documented who filled a prescription for one of the targeted medications: naltrexone, acamprosate, disulfiram, or topiramate. Intervention sites had 16 months of baseline pharmacy data prior to their first detailing contact, and were observed for 20 months thereafter. Mixed-effects segmented logistic regression [22] was used to estimate the parameters for the pre- and post-implementation segments, with random effects to account for the nesting of patients within months within sites. The first model, using the intervention sites as their own controls, compared the change in level from Month 16 in the pre-implementation period to the immediate post-implementation in Month 17 (i.e., “gap”) as well as the change in slope from the pre-implementation to the post-implementation period. In the second model, we compared the pre-post gap and changes in slope in the intervention sites to non-intervention control sites to ensure that the observed effects were not due to secular trends in the rest of VHA.

### Context and process factors

We then calculated and described facility-level variability in effectiveness, operationalized as the immediate pre-post implementation gaps and changes in slope. Then, we examined the context and process factors (i.e., moderators and mediators) that were associated with this variability and for which data were available from administrative sources or from Academic Detailing Program encounter logs. The main facility-level moderator examined was the pre-implementation rate of receipt of medications for AUD, calculated from VHA pharmacy data. Candidate mediators included total person-hours of detailing, total number of health care providers contacted by the program, and number of unique detailing sessions, all sourced from program encounter logs. We did not examine the effects of detailing encounter type (group vs. individual) or provider type on effectiveness due to substantial within-site variability on these factors. All candidate predictors were entered into the mixed-effect regression model described above, restricted to sites with detailing encounters, and interacted with the immediate gap and change in slope.

## Results

### Overall effectiveness in sites with detailing encounters

**Fig. 1:**
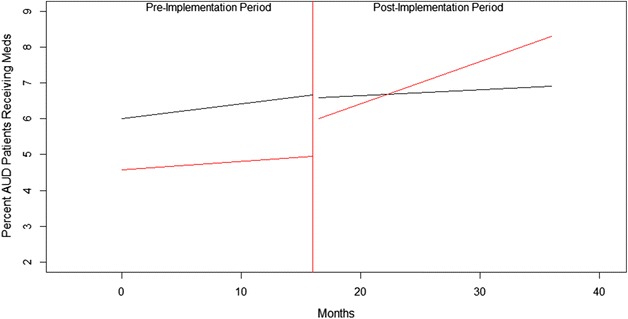
Effects of academic detailing program implemented in 37 VHA sites compared to secular trend in rest of VHA. The *red* segments represent the intervention sites and the *black* segments represent the control sites

For simplicity of interpretation, we converted the mixed-effects segmented logistic regression estimates into percents and graphically represented them in Fig. 1. As represented by the red trajectory in Fig. 1, the average proportion of patients with AUD who filled a prescription for naltrexone (oral or injectable), acamprosate, disulfiram, or topiramate in the 37 intervention sites was 4.56 % at baseline. The pre-implementation slope in the intervention sites was not significantly different than zero and increased to 4.96 % by Month 16. The gap of 1.04–6.00 % between Month 16 and 17 in the intervention sites was significant (p < .0001). Also, the pre-to-post increase in slope in the intervention sites compared to the pre-implementation slope was significantly positive (p < .0001), with the proportion of patients with AUD receiving medication increasing to 8.32 % by the end of Month 36. Thus from immediately pre-intervention to the end of the observation period (Month 16–Month 36), the percent of patients with AUD who received medication increased 3.36 % in absolute terms and 67.77 % in relative terms.

### Comparing medication trends in implementation and control sites

To check that these changes did not just mimic secular trends in the rest of VHA, we fitted a model that explicitly compared the four parameters in the intervention sites to the same parameters in all other VA sites. As represented by the black line in Fig. 1, at baseline in the rest of VHA, 6.01 % of patients with AUD filled a prescription for one of the medications, a proportion significantly higher than the intervention sites. The slope for the rest of VHA in the pre-intervention period was not significantly different than zero and increased to 6.67 % by the end of Month 16. The gap of −0.09 % between Month 16 and 17 in the rest of VHA to 6.58 % was slightly negative. The slope in the post-implementation period was slightly more positive than the pre-implementation period for the rest of VHA, ending at 6.90 % by the end of Month 36. Both the immediate gap and change in slope were significantly less than the intervention sites (p values <.001). Thus from Month 16 to Month 36 in the rest of VHA, the rate of medication receipt increased over 0.23 % in absolute terms and 3.45 % in relative terms.

### Context and process factors associated with program effects

**Fig. 2:**
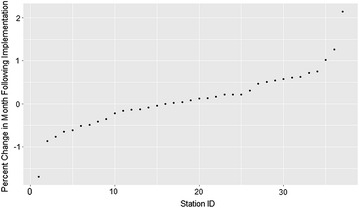
Variation in percent change in patients with AUD receiving medications in month following implementation

In addition to these overall effects, significant variability among the 37 sites in the effects of the Academic Detailing Program were found, with some sites having much bigger or smaller gaps and changes in slope than the overall average. Figure 2 shows the variation in the immediate post-implementation gaps for all 37 sites. Although the average gap was just over 1 %, it ranged from −1.69 to 2.14 %. The changes in slopes from the pre- to post-implementation period also varied, ranging from −0.55 to 0.08.

We then examined moderators and mediators that might explain variation in gaps and/or changes in slopes, as indicated by significant interaction terms in the mixed-effects segmented logistic regression. Descriptive statistics for these variables are presented in Table 2. Sites with lower pre-implementation adoption (i.e., lower proportion of patients receiving medications) had larger immediate increases in medication receipt after implementation (95 % confidence interval for interaction odds ratio 0.004–0.1114), as did sites with more person hours of detailing (95 % confidence interval for interaction odds ratio 1.00001–1.000187), but fewer people detailed (95 % confidence interval for interaction odds ratio 0.9904–0.9989). The average number of detailing encounters per person detailed was associated with steeper increases in slope over time (95 % confidence interval for interaction odds ratio 1.067–1.119).

**Table 2:** Descriptive statistics of site-level moderators and mediators of effectiveness

Variables	Mean (SD)	Range
Percent of AUD patients receiving medications	4.31 (2.54)	0–9.3
Person hours of detailing	26.63 (59.30)	0.15–330
Unique staff with detailing contact	31.76 (60.04)	1–325
Average encounters per detailed provider	1.12 (0.19)	1–1.8

## Discussion

In this study, we evaluated a multifaceted academic detailing program aimed at addressing a serious and persistent problem in healthcare quality—under-utilization of medications in the treatment of AUD. Despite strong evidence of clinical effectiveness of these medications, clinical and quality managers have thus far lacked evidence-based quality improvement strategies to increase access and utilization. Using a rigorous quasi-experimental design, we found that implementation of the Academic Detailing Program for AUD was associated with an overall immediate gap up in medication receipt as well a significant increase in the slope of adoption over time. From pre-intervention to the end of the follow-up period, the rate of medication receipt in the intervention sites increased approximately 3.4 % in absolute terms and 68 % in relative terms (i.e., 4.9–8.3 %). Although the intervention sites overall had lower levels of utilization at baseline compared to the rest of VHA, levels of utilization in the intervention sites were higher by the end of the observation period. Extrapolating this effect to the entire VHA would mean that over 11,000 additional patients with AUD would receive these effective medications. To our knowledge, this is the first quality improvement strategy focused on increasing access to AUD medications to be implemented on a large scale and shown to be effective overall.

Although the Academic Detailing Program increased prescribing of medications for treatment of AUD overall, the effect varied substantially across the intervention sites. In many sites, there was no improvement or even reductions in prescription fills. In an effort to explain some of this variability, we found that initially low performing facilities tended to improve the most. This result is encouraging and instructive. Although one might argue that those with lower baseline performance were more likely to improve due to regression to the mean [23], it is common in healthcare quality efforts for high performance sites to improve more than initially low performing sites. Also, facilities that received more detailing sessions and fewer people detailed tended to have the greatest initial improvement (biggest immediate gap) while larger increases in the slope of utilization over time were experienced in facilities that had a higher average number of detailing encounters per detailed person. In general, these results support the notion that more detailing produces more positive change, and suggests that perhaps focusing on a few key prescribers up front creates an immediate bump in prescribing while broader detailing encourages sustained growth in prescribing.

Several questions are left unanswered by this investigation. First, there were many aspects of the detailing encounters for which data were unavailable, including prior knowledge level of the target clinician as well as skill and experience of the detailer. Many facilities received detailing encounters from multiple detailers making the direct examination of detailer effects difficult. Also, one aspect of detailer skill and potential impact is the ability to secure initial meetings with facility staff. We had data on completed detailing encounters but not encounter requests. These are important limitations that should be addressed in future implementations and evaluations. Second, data on use of the informatics tools was not analyzed. Qualitative interviews with detailers and detailed clinicians might have helped explain why sites varied in response to the Academic Detailing Program, and may have provided perspectives and data that could be used to refine the program. Third, the analyses were based on filled prescriptions not on written prescriptions, for which no data were available. We expect the impact of this limitation to be minimal because we have no reason to believe that reliance on outside pharmacies or the proportion of unfilled prescriptions would be different in the pre and post implementation periods. Fourth, the campaign also sought to improve rates of screening, brief intervention, and referral to specialty treatment, data for which was unavailable for evaluation. Fifth, sustainment of effects after the program stopped was not examined. Sixth, data on cost of the intervention was not collected, precluding any budget impact or other economic analyses. Finally, long-term sustainment or emergence of effects after the detailing encounters stop were not evaluated but should be investigated in future analyses.

## Conclusions

Even with these limitations and caveats, this study is perhaps the first to find empirical support for a multifaceted quality improvement strategy aimed at increasing access to and utilization of effective medications in the treatment of AUD. Following the conclusion to this western VHA regional pilot for Academic Detailing for Mental Health Initiatives, in March 2015 VHA leadership requested the 21 regional networks to develop academic detailing programming to impact two other key medication safety quality improvement programs in VHA, the Opioid Safety Initiative and the Psychotropic Drug Safety Initiative. Future studies should rigorously evaluate the program’s effects on other quality improvement targets, focus on determining how to enhance the program effects, especially in sites where effects are weak. It is possible that less expensive and less labor intensive strategies would be adequate in some locations while even more intensive strategies are needed elsewhere. Learning how to identify the site characteristics that might be matched to the most efficient quality improvement strategy is the overarching goal of this line of research.

## Abbreviations

AUD: alcohol use disorder
US: United States
VHA: US Veterans Health Administration
FDA: US Federal Drug Administration
